# Pseudo-Benign Paroxysmal Positional Vertigo: A Retrospective Study and Case Report

**DOI:** 10.3389/fneur.2020.00187

**Published:** 2020-03-24

**Authors:** Jie Yu, Qianru Yu, Benling Guan, Yu Lu, Chengfang Chen, Shudong Yu

**Affiliations:** ^1^Department of Otolaryngology, Shandong Provincial Hospital Affiliated to Shandong University, Jinan, China; ^2^Shandong University of Tradition Chinese Medicine, Jinan, China; ^3^Department of Otolaryngology, Shandong Provincial Hospital Affiliated to Shandong First Medical University, Jinan, China

**Keywords:** pseudo-benign paroxysmal positional vertigo, vestibular migraine, valproic acid, canalith repositioning procedure, features, treatment

## Abstract

Pseudo-benign paroxysmal positional vertigo (pseudo-BPPV) is a specific type of vestibular migraine disguised as benign paroxysmal positional vertigo, which is characterized by recurrent different types of positional and atypical positional vertigo with migraine features. It is easy to be misdiagnosed with BPPV at the first visit, which means that the ideal therapeutic effects are not achieved. Twenty-five cases of pseudo-BPPV with frequent changing positional vertigo were retrospected and the following key features help to identify the disease: recurrent positional and atypical positional vertigo, migrainous accompanying symptoms or migraine history, mild or indistinctive headaches, with or without impaired vestibular function, ineffective for simply reposition. And we found that vertigo in pseudo-BPPV can be preferable controlled by valproic acid combined with canalith repositioning procedure.

## Introduction

Vestibular migraine (VM) as a distinct diagnostic term has been defined by the Bárány Society and the International Headache Society (IHS) (1). It is identified as a condition that consists of vestibular symptoms and migraine. Symptoms of vestibular migraine are often indistinguishable from symptoms of dizziness caused by other pathogenesis, such as benign paroxysmal positional vertigo (BPPV) (2). Positional vertigo was first described by Robert Barany in 1921, and the term BPPV was coined by Dix and Hallpike (3). BPPV is characterized by brief attacks of rotatory vertigo associated with positional and/or positioning nystagmus, which are elicited by changes in the head position relative to gravity (4). There are several reports that some patients with VM can experience frequent episodes of BPPV (2, 5, 6). von Brevern et al. (5) observed 10 patients with VM whose symptoms were very similar to BPPV and named the disease pseudo-BPPV. They distinguished migrainous positional vertigo from BPPV and proposed diagnostic criteria for pseudo-BPPV, but lacked of relevant research on features and treatment of pseudo-BPPV. The aim of the present report was to explore the clinical characteristics and therapeutic options of pseudo-BPPV to aid normative diagnosis and treatment.

## Materials and Methods

We retrospectively recruited 25 patients form 458 patients with certain characteristics of VM and BPPV from our outpatient and ward according to a protocol approved by the hospital ethics committee. All of the 25 patients have written informed consent for this study. These patients had experienced recurrent short-duration vertigo caused by postural changes and accompanied with migraine features from August 2016 to January 2019 in ENT department of Shandong Provincial Hospital affiliated to Shandong University and the first Hospital affiliated to Shandong First Medical University. Eligible participants were diagnosed with pseudo-BPPV based on the following diagnostic criteria (5, 7).

(1) Episodic positional vertigo with a duration of <1 min, and positional vertigo attacks continued after successful repositioning maneuver. (2) Positional vertigo attacks with accompanying migrainous symptoms (headache, photophobia, phonophobia, visual, or other aura) or migraine history. (3) According to the Bárány Society and the IHS criteria, symptoms met the diagnostic criteria of definite VM or probable VM (see Table 1) (7, 8). (4) Exclude vertigo from other causes by appropriate investigations.

**Table 1:** Diagnostic criteria for definite VM and probable VM.

1. Definite VM
A. At least 5 episodes with vestibular symptoms of moderate or severe intensity, lasting 5 min to 72 h
B. Current or previous history of migraine with or without aura according to the International Classification of Headache Disorders (ICHD)
C. One or more migraine features with at least 50% of the vestibular episodes:
- headache with at least two of the following characteristics: one sided location, pulsating quality, moderate or severe pain intensity, aggravation by routine physical activity
- photophobia and phonophobia
- visual aura
D. Not better accounted for by another vestibular or ICHD diagnosis
2. Probable VM
A. At least 5 episodes with vestibular symptoms of moderate or severe intensity, lasting 5 min to 72 h
B. Only one of the criteria B and C for vestibular migraine is fulfilled (migraine history or migraine features during the episode)
3. Other causes ruled out by appropriate investigations

All patients received physical otolaryngology examination and magnetic resonance imaging (MRI) of the brain and internal auditory canal. In the meantime, electronystagmography (ENG) was recorded to assess vestibular functions were impaired. High stimulus rate auditory brainstem responses (ABRs) were also recorded to identify posterior circulation ischemia. All patients with pseudo-BPPV immediately underwent the relevant canalith repositioning procedure once a day until the position vertigo and nystagmus disappeared in positioning test and experimental application of valproic acid (Depakin, 500 mg, Sanofi, France) for the entire 1 month treatment period. They then stopped taking the medicine and we observed the effects on disease control at 6 months.

## Case Report

A 74-year-old woman (informed consent has been signed) with a long history of vertigo, reported experiencing her first vertigo attack lasting for half an hour two decades ago, without any symptoms of tinnitus and deafness. Moderate unilateral headache occurred during the vertigo attack. The vertigo episode was not related to her body position, and was resolved without any treatment. The patient then frequently experienced vertigo three to four times per year. About 10 years ago, the frequency of rotatory dizziness increased to 20 to 30 times per day, and the vertigo was often accentuated by postural changes especially during sleep. The postural vertigo had a short duration of approximately 10 s. Migraine headaches without any aura since the age of 42 years. She denied history of other types of aura, tinnitus or hearing loss. The patient had no previous history of other diseases. Her family history was unremarkable except for migraine headaches in her mother.

**Figure 1:**
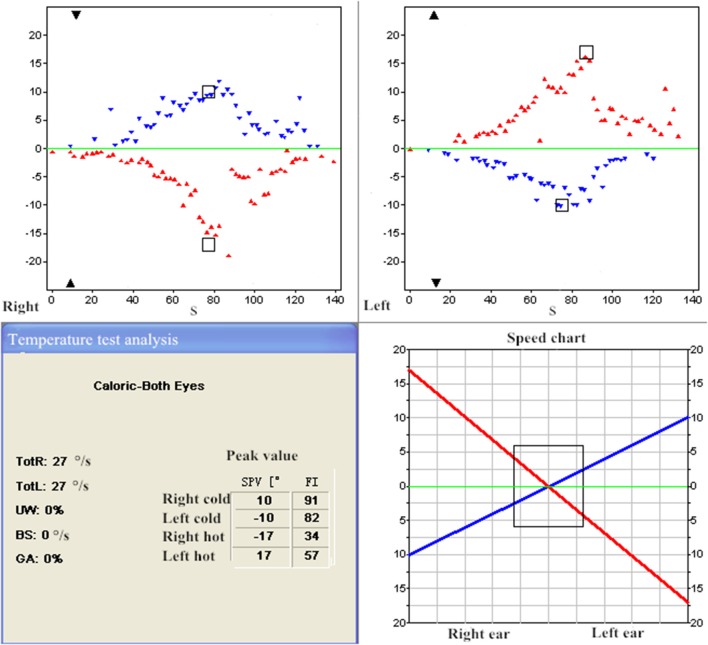
ENG in the patient with pseudo-BPPV (patient 2). UW = 0% < 25% revealed normal vestibular functions.

**Figure 2:**
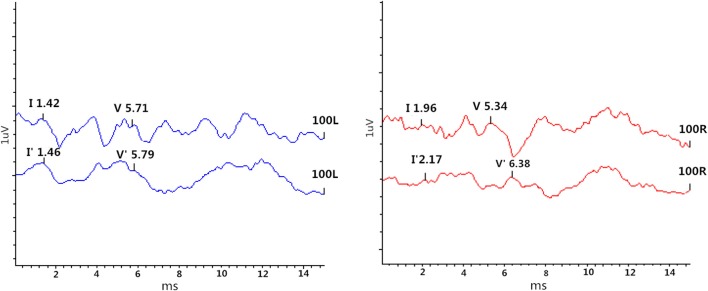
High stimulus rate ABRs in the patient with pseudo-BPPV (patient 2). Left ear: (V'-I')–(V-I) = 0.04 ms < 0.28 ms revealed normal; Right ear: (V'-I')–(V-I) = 0.83 ms > 0.28 ms revealed abnormal.

Before she came to our clinic, she had been diagnosed with BPPV in several hospitals. The canalith repositioning procedure was effective, but vertigo recurred quickly. She was first evaluated in our clinic during a vertigo episode. Geotropic nystagmus was seen in the Dix-Hallpike test. It was induced at the right vertical suspension head position and persisted for 10–15 s accompanied by drastic vertigo. She tested negative for the Romberg's sign. No Spontaneous and gaze-evoked nystagmus were caught in the clinical test. Normal ocular alignment, ocular versions and convergence. We performed the standard Epley manual reduction and the nystagmus disappeared about 2 days. Dizziness followed by nausea reappeared while she was turning to the left supine decubitus position from the supine position. Left-beating nystagmus was found in the lateral roll test and soon disappeared after Barbecue manual reduction. Multiple similar episodes frequently occurred. After each repositioning maneuver, vertigo still has a relapse. She recalled pulsatile headache aura before the latest episodes of vertigo. ENG revealed no evident abnormal vestibular function (Figure 1). High stimulus rate ABRs revealed abnormality in the right side (Figure 2). High-resolution computed tomography of the temporal bone revealed unremarkable results associated with vertigo. Brain and internal auditory canal MRI was performed for possible vertebrobasilar insufficiency, no structural lesions were found in the internal auditory canal or brain. Except for the canalith repositioning procedure once a day until the position vertigo and nystagmus disappeared in position test, we administered experimental treatment with valproic acid (Depakin, 500 mg, Sanofi, France, 0.5 g twice a day) for 1 month. After 1 week of treatment, the position vertigo disappeared. At the 6 month follow-up, she reported no persistent vertigo, headache aura, or positional nystagmus episodes.

## Results

In the 458 patients with certain characteristics of VM and BPPV in our department, twenty-five patients were identified and diagnosed as a variant of vestibular migraine: pseudo-BPPV. A total of 25 patients aged 27 to 85 years were referred to our research (Table 2). All patients met the inclusion criteria. Female patients accounted for 72% of the sample, showing a higher morbidity in women. We divided the patients we collected into two groups: patients with accompanying migrainous symptoms and patients with migraine history. Among 25 patients suffering from pseudo-BPPV, 18 patients had migraine features before or during episodes. Seven of these 18 patients had headaches, five patients felt fullness in the head, four patients had phonophobia, two patients had photophobia and one patient had visual aura. The other 7 patients of these 25 patients denied experiencing any migraine features associated with vertigo during their attacks, but recalled a previous history of migraine. The attack frequency of rotational dizziness varied from 1 to 30 times per day. Each positional vertigo episode lasted from 2 to 3 s to 1 min. Throughout the entire course of the disease, we found that in addition to positional vertigo lasting <1 min, patients also experienced episodes of non-positional vertigo lasting more than 1 min, this usually lasted several minutes and met the diagnostic criteria for VM or probable VM (not listed into the Table 2). Different types of nystagmus were seen in positioning test, we found the positive posture changed in 24 patients, diverse nystagmus was observed during different attacks, and only one patient's nystagmus direction remained unchanged. Four of the 25 patients showed ENG abnormalities and 8 patients showed abnormalities in high stimulus rate ABRs. MRI revealed no organic lesions in the internal auditory canal, cerebrum, brainstem, or cerebellum in any patients.

**Table 2:** Clinical details of patients with pseudo-BPPV.

**Patient No./The age range**	**Positioning test**	**Attack frequency per day (times)**	**Duration of each positional vertigo**	**Migraine features during episodes^**#**^**	**Nystagmus seen in the positioning test for the first time**	**Responding to valproic acid combined with repositioning maneuver**	**ENG/High stimulus rate ABRs**	Relapse*
**PATIENTS WITH ACCOMPANYING MIGRAINOUS SYMPTOMS**								
1/25–30	No change	2–3	<1 min	Headache	Geotropic nystagmus in the bilateral roll test	Effective	–/–	Not reoccurred
2/70–75	Change	20–30	10–15 s	Headache	Geotropic nystagmus in the right Dix-Hallpike test	Effective	–/+	Not reoccurred
3/75–80	Change	4–5	10 s	Fullness in head	Geotropic nystagmus in the bilateral roll test	Effective	–/–	Not reoccurred
4/30–35	Change	1–2	1 min	Headache	Apogeotropic nystagmus in the left roll test	Effective	–/+	Not reoccurred
5/40–45	Change	3–4	1 min	Fullness in head	Atypical nystagmus in the right Dix-Hallpike test	Effective	+/+	Not reoccurred
6/40–45	Change	1–2	30 s	Photophobia	Atypical nystagmus in the right Dix-Hallpike test	Effective	+/–	Not reoccurred
7/80–85	Change	10–15	3–4 s	Headache	Apogeotropic nystagmus in the bilateral roll test	Effective	–/–	Not reoccurred
8/60–65	Change	2–3	<10 s	Fullness in head	NA	Effective	–/–	Not reoccurred
9/50–55	Change	2–3	20–30 s	Phonophobia	Geotropic nystagmus in the left Dix-Hallpike test	Effective	–/–	Not reoccurred
10/65–70	Change	2–3	10–20 min	Phonophobia	NA	Effective	–/–	Not reoccurred
11/40–45	Change	1–2	<1 min	Fullness in head	NA	Effective	–/–	Not reoccurred
12/55–60	Change	5–6	4–5 s	Fullness in head	Geotropic nystagmus in the right Dix-Hallpike test	Effective	–/–	Not reoccurred
13/40–45	Change	10–20	<10 s	Headache	Geotropic nystagmus in the left Dix-Hallpike test	Effective	–/–	Not reoccurred
14/60–65	Change	1–2	5–6 s	Headache	Geotropic nystagmus in the left roll test	Effective	–/–	Not reoccurred
15/55–60	Change	1–2	7–8 s	Phonophobiaphotophobia	Apogeotropic nystagmus in the bilateral roll test	Effective	+/+	Reoccurred
16/50–55	Change	3–4	15 s	Headache	NA	Effective	+/+	Reoccurred
17/60–65	Change	7–8	<10 s	Phonophobia	NA	Ineffective	–/–	–
18/55–60	Change	5–6	<10 s	Visual aura	NA	Ineffective	–/–	–
**PATIENTS WITH MIGRAINE HISTORY**								
19/70–75	Change	1–2	<10 s	None	Apogeotropic nystagmus in the bilateral roll test	Effective	–/–	Not reoccurred
20/55–60	Change	1–2	4–5 s	None	Geotropic nystagmus in the left Dix-Hallpike test	Effective	–/–	Not reoccurred
21/45–50	Change	8–9	<10 s	None	NA	Effective	–/–	Not reoccurred
22/75–80	Change	3–4	1 min	None	NA	Effective	–/+	Not reoccurred
23/45–50	Change	1–2	3–4 s	None	Atypical nystagmus in the roll test and Dix-Hallpike test	Effective	–/–	Not reoccurred
24/70–75	Change	2–3	7–8 s	None	Geotropic nystagmus in the right Dix-Hallpike test	Effective	–/+	Not reoccurred
25/75–80	Change	7–8	<1 min	None	NA	Effective	–/+	Reoccurred

Relapse*: During half a year follow-up, whether the vertigo had reoccurred after the patients stopping taking the valproic acid.

*Migraine features during episodes^#^: The term headache in this column refer to qualitative characteristics of migraine: headache with at least two of the following characteristics: one sided location, pulsating quality, moderate or severe pain intensity, aggravation by routine physical activity*.

*Ineffective: The frequency and severity of vertigo did not decrease within a month after taking the valproic acid*.

After treatment with valproic acid (Depakin, 500 mg, Sanofi, France, 0.5 g twice a day) combined with the relevant canalith repositioning procedure, two patients experienced no significant benefit and the remaining 23 patients experienced a relief in their symptoms in 1 month and then stopped taking the medication. At the 6 month follow-up, 20 patients had a significant decrease in vertigo frequency and reported no recurrence. Three patients relapsed and were treated with appropriate repositioning maneuvers combined with valproic acid again, and then their vertigo also disappeared.

## Discussion

In our study, we identified a group of patients who had suffered from frequent short-duration attacks of vertigo with accompanying migrainous symptoms or migraine history. This study was promoted by our clinical experience, we aimed to retrospectively identify the characteristics of pseudo-BPPV, distinguish it from BPPV to reduce misdiagnosis, and discuss the treatment for it.

Increasing recent evidence has highlighted the association between migraine and BPPV (9, 10). Episodic positional vertigo is the typically manifestation of BPPV (2). BPPV can be classified into primary and secondary BPPV. In most cases, the cause of BPPV is not evident. BPPV can also be secondary to other diseases including VM (5). The Dix-Hallpike and Roll maneuvers are used to diagnose BPPV which can be established by anamnesis and detection of positional nystagmus (11). Simple otolithiasis can be cured clinically by canalith repositioning procedure. The success rate of canalith repositioning procedure for different types of BPPV is 60.9–90%, and repeated treatments can improve the success rate (12). By contrast, typical symptoms of VM can be triggered along with spontaneous vertigo, associated with phonophobia and photophobia, nausea, aural symptoms, and headaches (13). Damage to the otolith organs may cause positional vertigo, but there is generally no positional nystagmus in VM. Pseudo-BPPV is a complex mix of positional, atypical positional and non-positional vertigo accompanied by migraine features. It is actually a combination of BPPV and frequent short-duration VM episodes. The ability to distinguishing pseudo-BPPV from other vertigo disease has great clinical significance for treatment.

Our study collected 25 cases basically met the diagnostic criteria of pseudo-BPPV. Because the diagnostic criteria of VM have been updated in 2012 (7), we also updated the criteria of pseudo-BPPV. In our study, the majority of patients showed normal vestibular functions (according to ENG results) and normal posterior circulation (according to high stimulus rate ABRs), but it seemed to have no guidance for the diagnosis of the disease. According to their medical history, most patients had different accompanying symptoms such as migraine aura, visual or auditory signs (blurred vision, afraid of bright lights or irritated by noises during vertigo attacks). These signs were collectively considered as migraine features (based on the IHS criteria). It is worth mentioning that headaches in pseudo-BPPV are not as intense or typical as migraine, and some patients described their headaches as a feeling of fullness in the head. In addition, the patients with previous history of migraine but lack of accompanying migrainous symptoms during vertigo could also be included into pseudo-BPPV, because these patients met the diagnosis of probable VM. Throughout the course of the disease, the patients experienced episodes consistent with typical VM, as well as episodes similar to BPPV. In the present of 25 patients, recurrent positional vertigo lasted <1 min, while non-positional spontaneous vertigo lasted for several minutes to hours. Because the characteristics of accompanying migrainous symptoms and previous migraine history met the features for VM, we considered pseudo-BPPV to be a special type of VM. The characteristic of positional vertigo and shorter duration in patients with pseudo-BPPV were similar to that of BPPV, so it was easy to be misdiagnosed.

When positional vertigo is mixed in the vertigo attacks of patients with VM and accounted for the major symptom, it is easy to be misdiagnosed with BPPV. Furthermore, the medical history and concomitant symptoms at the time of patients' first visit are often overlooked. Therefore, when a patient who has previously been diagnosed with VM or migraine headaches in the past presents with frequent short-duration positional vertigo similar to BPPV, the possibility of pseudo-BPPV should be considered.

There are some notable differences between pseudo-BPPV and BPPV. Episodes of pseudo-BPPV are often characterized by migraine features, whereas BPPV is characterized by positional vertigo without any accompanying migraine symptoms. Positional vertigo is not as typical in pseudo-BPPV as in BPPV. Nystagmus of a patient with pseudo-BPPV is sometimes inconspicuous and can change frequently, whereas nystagmus in a patient with BPPV is normally severe and in a fixed position. Although the duration of each episode induced by positional changes is similar to that of BPPV (<1 min), the duration of the entire course of the disease is different: vertigo in BPPV typically last weeks to months until spontaneous ease (5), which is determined by the natural metabolic time of the otoliths, whereas in pseudo-BPPV, the majority of patients have a longer history of vertigo.

Actually, there were two types of position vertigo in the patients with pseudo-BPPV. First, patients sometimes have vertigo attacks when the position changes (such as turning the head), but the vertigo and nystagmus induced in the position of Dix-Hallpike test or lateral roll test are atypical. Second, position vertigo and nystagmus can be induced by positioning test as typical as BPPV. Canalith repositioning maneuver can relief this type of vertigo to some extent. The common features for these two types are that the positional vertigo accompany with migraine features and the duration is <1 min. To be specific, the first type is the episode of short-duration vertigo of VM, the second type is the episode of secondary BPPV induced by VM. The 25 patients we collected were not simply a combination of VM and BPPV. The two diseases co-existed, and manifested as a combination of short period attack of VM and recurrent BPPV, called pseudo-BPPV.

Canalith repositioning procedure was first performed for positional vertigo in patients with pseudo-BPPV. This procedure temporarily relieved vertigo, but vertigo quickly recurred for the existence of VM. We came to realize that this was not a simple episode of BPPV, because in typical BPPV, vertigo can be relieved continuously in most cases. In these 25 patients, after many times of successful repositioning maneuver, vertigo still recurred. Then, except for repositioning maneuver once a day, we administered valproic acid to prevent the occurrence of BPPV that secondary to VM. Valproic acid, also known as sodium valproateis, is the first-line clinical treatment of systemic and partial seizure epilepsy (14) and absence seizures in children and adolescents (15). It is also administered for the treatment of migraine headaches (16). The main mechanism of valproic acid may be related to indirectly regulating neurotransmitter release through the regulation of ionic currents and facilitating GABAergic over glutamatergic transmission (14). Previous studies have shown that valproic acid more effectively decreased the frequency of headaches and vestibular symptoms in VM than other drugs (17, 18). The characteristics of short-duration, high frequency and migraine features in pseudo-BPPV just fit the indications of valproic acid mentioned above. In order to evaluate the effect, we used the same dose which is the lowest dose (500 mg twice a day). Our results suggested that the effects of valproic acid combined with canalith repositioning were evident after 1-month treatment, and this treatment could control recurrence of the disease to some extent. We observed that most people had good effect with the lowest dose, and reuse of valproic acid is still effective for recurrence of vertigo. In addition, different types of medications have been typically used in VM prophylaxis including anticonvulsants (topiramate), calcium antagonists (flunarizine), β-blockers (propranolol and metoprolol), and antidepressants (amitriptyline and venlafaxine) (16, 19–21). In our earlier study, we found that most drugs (calcium antagonists, β-blockers and antidepressants) had no significant effect in treating pseudo-BPPV. Whether other antiepileptic drugs have the same effect requires further observation.

In summary, pseudo-BPPV is a particular type of VM disguised as BPPV. It has some key features: recurrent positional and atypical positional vertigo, accompanying migrainous symptoms or migraine history, mild or indistinctive headache, with or without impaired vestibular function, ineffective for simply reposition but effective for reposition combined with valproic acid. The disease may also have related family history, and the incidence is higher in women.

The present study has some limitations. The number of cases included in the study was not large enough. The pathogenesis of pseudo-BPPV is still a matter of speculation. The mechanism of action, dosage and best medication time of valproic acid need to be further studied. In addition, retrospective study have some limitations, randomized controlled trials are needed in the future.

## Data Availability Statement

All datasets generated for this study are included in the article/supplementary material.

## Ethics Statement

Written informed consent was obtained from the individual(s) for the publication of any potentially identifiable images or data included in this article.

## Author Contributions

JY participated in the entire retrospective study, patient follow-up, data collection, and manuscript writing. SY conducted this retrospective study design and case collection. QY participated in case collection. BG and YL participated in patient follow-up and documentation. CC participated in the search of relevant literature.

### Conflict of Interest

The authors declare that the research was conducted in the absence of any commercial or financial relationships that could be construed as a potential conflict of interest.

## References

[1] von Brevern M Lempert T. Vestibular migraine. Handb Clin Neurol. (2016) 137:301–16. 10.1016/B978-0-444-63437-5.00022-4 27638080

[2] Beh SC. Horizontal direction-changing positional nystagmus and vertigo: a case of vestibular migraine masquerading as horizontal canal BPPV. Headache. (2018) 58:1113–7. 10.1111/head.13356 30152162

[3] Dix MR Hallpike CS. The pathology symptomatology and diagnosis of certain common disorders of the vestibular system. Proc R Soc Med. (1952) 45:341–54. 10.1177/003591575204500604 14941845 PMC1987487

[4] Imai T Takeda N Ikezono T Shigeno K Asai M Watanabe Y. Classification, diagnostic criteria and management of benign paroxysmal positional vertigo. Auris Nasus Larynx. (2017) 44:1–6. 10.1016/j.anl.2016.03.013 27174206

[5] von Brevern M Radtke A Clarke AH Lempert T. Migrainous vertigo presenting as episodic positional vertigo. Neurology. (2004) 62:469–72. 10.1212/01.WNL.0000106949.55346.CD 14872034

[6] Neuhauser H Leopold M von Brevern M Arnold G Lempert T. The interrelations of migraine, vertigo, and migrainous vertigo. Neurology. (2001) 56:436–41. 10.1212/WNL.56.4.436 11222783

[7] Lempert T Olesen J Furman J Waterston J Seemungal B Carey J. Vestibular migraine: diagnostic criteria. J Vestib Res. (2012) 22:167–72. 10.3233/VES-2012-0453 23142830

[8] Lempert T Olesen J Furman J Waterston J Seemungal B Carey J. [Vestibular migraine: diagnostic criteria: consensus document of the Barany Society and the International Headache Society]. Nervenarzt. (2013) 84:511–6. 10.1007/s00115-013-3768-x 23532572

[9] von Brevern M Neuhauser H. Epidemiological evidence for a link between vertigo and migraine. J Vestib Res. (2011) 21:299–304. 10.3233/VES-2011-0423 22348934

[10] Pollak L Pollak E. Headache during a cluster of benign paroxysmal positional vertigo attacks. Ann Otol Rhinol Laryngol. (2014) 123:875–80. 10.1177/0003489414539921 25015924

[11] Evren C Demirbilek N Elbistanli MS Köktürk F Çelik M. Diagnostic value of repeated dix-hallpike and roll maneuvers in benign paroxysmal positional vertigo. Braz J Otorhinolaryngol. (2017) 83:243–8. 10.1016/j.bjorl.2016.03.007 27170347 PMC9444768

[12] You P Instrum R Parnes L. Benign paroxysmal positional vertigo. Laryngoscope Investig Otolaryngol. (2019) 4:116–23. 10.1002/lio2.230 30828628 PMC6383320

[13] Beh SC Masrour S Smith SV Friedman DI. The spectrum of vestibular migraine: clinical features, triggers, and examination findings. Headache. (2019) 59:727–40. 10.1111/head.13484 30737783

[14] Romoli M Mazzocchetti P D'Alonzo R Siliquini S Rinaldi VE Verrotti A. Valproic acid and epilepsy: from molecular mechanisms to clinical evidences. Curr Neuropharmacol. (2018) 17:926–46. 10.2174/1570159X17666181227165722 30592252 PMC7052829

[15] Brigo F Igwe SC Lattanzi S Ethosuximide, sodium valproate or lamotrigine for absence seizures in children and adolescents. Cochrane Database Syst Rev. (2019) 2:D3032 10.1002/14651858.CD003032.pub4 PMC6367681 30734919

[16] Karimi N Razian A Heidari M. The efficacy of magnesium oxide and sodium valproate in prevention of migraine headache: a randomized, controlled, double-blind, crossover study. Acta Neurol Belg. (2019). 10.1007/s13760-019-01101-x. [Epub ahead of print]. 30798472

[17] Celiker A Bir LS Ardiç N. Effects of valproate on vestibular symptoms and electronystagmographic findings in migraine patients. Clin Neuropharmacol. (2007) 30:213–17. 10.1097/wnf.0b013e31803bb3ee 17762318

[18] Liu F Ma T Che X Wang Q Yu S. The efficacy of venlafaxine, flunarizine, and valproic acid in the prophylaxis of vestibular migraine. Front Neurol. (2017) 8:524. 10.3389/fneur.2017.00524 29075232 PMC5641552

[19] Obermann M Strupp M. Current treatment options in vestibular migraine. Front Neurol. (2014) 5:257. 10.3389/fneur.2014.00257 25538676 PMC4255594

[20] Bisdorff AR. Management of vestibular migraine. Ther Adv Neurol Disord. (2011) 4:183–91. 10.1177/1756285611401647 21694818 PMC3105632

[21] Maldonado Fernández M Birdi JS Irving GJ Murdin L Kivekäs I Strupp M Pharmacological agents for the prevention of vestibular migraine. Cochrane Database Syst Rev. (2015) 2015:D10600 10.1002/14651858.CD010600.pub2 PMC6494480 26093662

